# The problem with one-size-fits-all medicine: Biological sex and the aging immune system

**DOI:** 10.1371/journal.pbio.3003763

**Published:** 2026-05-11

**Authors:** Clayton Baker, Victor A. Ansere, Cossette I. Sanqui, Bérénice A. Benayoun

**Affiliations:** 1 Leonard Davis School of Gerontology, University of Southern California, Los Angeles, California, United States of America; 2 Molecular and Cellular Biosciences Department, USC Dornsife College of Letters, Arts and Sciences, Los Angeles, California, United States of America; 3 Cancer Biology Department, Keck School of Medicine University of Southern California, Los Angeles, California, United States of America; 4 Pharmacology and Pharmaceutical Sciences Department, USC Mann School of Pharmacy and Pharmacology, Los Angeles, California, United States of America; 5 USC Norris Comprehensive Cancer Center, Epigenetics and Gene Regulation, Los Angeles, California, United States of America; 6 USC Stem Cell Initiative, University of Southern California, Los Angeles, California, United States of America; Harvard University T H Chan School of Public Health, UNITED STATES OF AMERICA

## Abstract

Aging has effects on the immune system that are similar in men and women, but also reshapes their immune systems in unique, sex-specific ways. These sex-specific patterns of immune aging influence disease susceptibility, vaccine effectiveness, cancer survival, and responses to pharmacological therapies, and have direct implications for preventive medicine and clinical care. However, these differences in susceptibilities and responses are rarely considered in research, clinical trials, or treatment guidelines. By integrating knowledge of sex-specific immune aging with real-world outcomes from vaccines, cancer immunotherapy, and pharmacovigilance studies, this Essay argues that accounting for both sex and age is essential to advance personalized medicine.

## Abstract 2

Aging has effects on the immune system that are similar in men and women, but also reshapes their immune systems in unique, sex-specific ways, however these differences are rarely taken into consideration. This Essay argues that accounting for both sex and age is essential to advance personalized medicine.

## Introduction

The immune system can be divided into two categories: innate and adaptive [1]. Innate immune cells (e.g., neutrophils, macrophages, and dendritic cells [DCs]) release cytokines and pro-inflammatory mediators that coordinate the immune response and protect the host [2]. By contrast, the adaptive immune system provides a targeted and long-term defense against pathogens [3]. While innate responses are rapid and general, adaptive immunity is slower and highly specific [3]. A hallmark of adaptive immunity is immunological memory, which provides an effective, tailored response to previously encountered pathogens [3]. T cells, B cells, and antibodies (produced by B cells) work together to provide this specificity and memory. Importantly, innate and adaptive immunity are interconnected, constantly communicating to maintain immune balance and protection [4]. For example, helper T cells stimulate B cells to undergo immunoglobulin class switching, producing IgE antibodies that activate innate immune cells [4]. This triggers the release of pro-inflammatory mediators (e.g., histamines, proteases, cytokines, and chemokines), generating an immediate response [4]. Similarly, antigen-presenting cells of the innate immune system (e.g., macrophages and DCs) activate pathogen-specific T cells and B cells by processing antigens, displaying peptide–MHC complexes, and delivering co-stimulatory and cytokine signals [4]. Through these interactions, the innate and adaptive systems coordinate a highly effective immune defense.

Like other biological systems, the immune system undergoes age-related functional decline [5]. Indeed, the latest hallmarks of aging recognized by the field now include chronic inflammation as a distinct hallmark, recognizing its crucial role in aging phenotypes [6]. Changes in the immune system can both promote or restrain aging across multiple organs [6]. Two main characteristics of immune aging are ‘immunosenescence’ and ‘inflammaging’ (Box 1) [7]. Together, these processes promote the development of age-associated diseases (e.g., atherosclerosis, dementia, osteoarthritis) [7]. Understanding hallmarks of immune aging is critical, as they influence both life span and healthspan (Box 1) [8].

**Box 1. Glossary.**

**Immunosenescence**

The age-related decline in immune system function.

**Inflammaging**

Chronic, sterile inflammation that occurs with age, even without infection.

**Sex differences**

The difference in form or function between sexes of the same species.

**Healthspan**

The number of years lived in good health.

**Vaccine immunogenicity**

The strength of a vaccination-induced immune response.

**Clinical pharmacology**

The study of how drugs behave in humans.

**Maternal-fetal microchimerism**

Crossing of cells between the placenta and mother during pregnancy, which remain in both mother and fetus.

In addition to shared age-related changes, sex differences further shape immune aging [9]. Sex differences (Box 1) lead to divergent patterns of life span and healthspan between males and females [10]. Overall, females tend to live longer than males, yet experience more age-related and immune-related diseases, whereas males are more likely to develop severe outcomes from infections [10,11]. This discrepancy, in which females outlive males but spend more years in poor health, is referred to as the ‘morbidity-mortality’ paradox [10]. One potential driver of sex differences in disease susceptibility and health outcomes is maternal-fetal microchimerism (Box 1), which has been shown to modulate the immune system [12]. However, sex differences in disease susceptibility and health outcomes are thought to be mainly driven by the effect of sex chromosomes (XX versus XY) and/or sex hormones on the immune system [10].

Indeed, the X chromosome contains many immune genes, some of which escape X inactivation with aging, contributing to stronger immune responses in females [9]. By contrast, the Y chromosome encodes relatively few immune genes, which contributes to sex differences in immune system robustness [9]. Reflecting this difference in copy number, females generally produce more cytokines than males regardless of age [9]. Sex-steroid receptors are expressed broadly in immune cells, though absolute levels vary [9]. Estrogens exert both pro-inflammatory and anti-inflammatory effects, depending on concentrations, whereas androgens suppress immune activity [9,13,14]. Thus, across the female life span, fluctuations in estrogen levels with menstrual cycling, pregnancy, and menopause influence immune function, with menopause associating with increased susceptibility to autoimmunity [9]. In males, age-related androgen decrease can impact immune function by promoting inflammation and altered responses to infections and vaccines [15]. Together, molecular and hormonal differences have functional consequences, shaping differences in disease susceptibility, vaccine responses, and infection resilience, ultimately leading to different aging trajectories across sexes.

With aging, females maintain adaptive immune responses more effectively than males, suggesting that the female immune system has higher baseline activity, with stronger expression of adaptive versus innate immune pathways [9]. Conversely, aging males rely more heavily on innate immunity, which may partly explain heightened innate responses but poorer outcomes following infections and vaccinations [9]. This sex difference may be due to overall higher levels of testosterone in males, which has been shown to have important impacts the immune system over time [14]. However, while stronger immune responses provide protection in females, they also increase autoimmunity risk with age [13]. Sex differences in immune aging highlight how differences in both adaptive and innate immunity shape lifelong susceptibility to infections and age-related diseases, emphasizing the importance of sex as a biological variable in both immunological research and clinical care.

The profound influence of both age and sex on immunity underscores the importance of incorporating these variables into research and clinical practice. Yet, many continue to overlook the biological impacts of sex. In this Essay, we argue that considering sex, age, and how they interact is essential for advancing personalized medicine and improving health outcomes for all. Although gender may influence lifelong immunity through sociocultural and behavioral factors [9], our discussion is confined to biological sex-based mechanisms that drive differences in immune aging. Throughout this Essay, we integrate insights into how sex, age, and their often nonlinear interaction shape the immune system with real-world outcomes from disease incidence and severity, vaccines, cancer immunotherapy, and pharmacovigilance studies to underscore the importance of considering both sex and age in advancing personalized medicine.

## How does sex affect immune aging?

**Fig 1:**
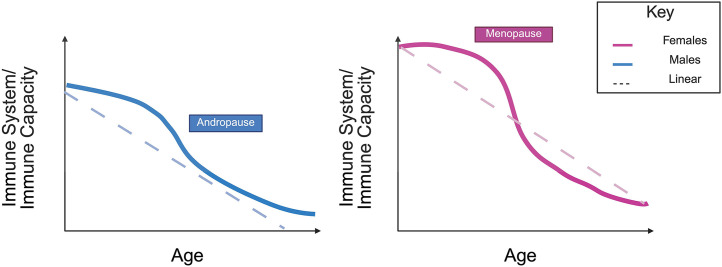
Nonlinear effects of sex on immune aging. Concept illustrating sex-specific, nonlinear trajectories of immune capacity across the life span. Immune function declines with age in both females (magenta) and males (blue), but differs in timing and slope. In females, immune aging is depicted with a pronounced inflection around menopause, corresponding to abrupt hormonal changes, whereas in males, immune decline is depicted as more gradual, consistent with progressive andropause. Dashed lines represent simplified linear models that fail to capture these sex-by-age dynamics, underscoring the importance of considering nonlinearity in studies of immune aging. This figure was created using BioRender. Baker, C. (2025) https://BioRender.com/ky5yq9h.

The immune system differs between sexes, and the evolution of these differences with aging is nonlinear (Fig 1) [16]. Sex shapes the immune system in complex, dynamic ways [17]. Some differences are constant throughout life, while others will change, and early-life differences may weaken with old age as overall competence declines [16,17]. This nonlinearity is a crucial factor in understanding and addressing immune aging in both sexes. Ignoring this interplay has led to the production of therapies optimized for neither sex nor age that fail when immune resilience is most critical.

### Underlying biological factors

As individuals age, both innate and adaptive immunity decline, leading to weaker vaccine responses, reduced pathogen clearance, and heightened inflammation following viral infections [5]. These changes are not uniform across individuals but are strongly influenced by sex-specific hormone trajectories [18,19]. Although older adults consistently exhibit increased amounts of pro-inflammatory cytokines and activated pro-inflammatory gene programs, pronounced sex differences in these features remain relevant clinical factors [20–22].

Menopause in women, defined as the cessation of ovarian function, results from depletion of ovarian follicles and the consequent decline in circulating estrogen levels [23,24]. Beyond its reproductive correlates, menopause is a biological waypoint that reshapes immune function and, consequently, the body’s response to immunotherapy [19]. For example, distinct shifts in T-cell dynamics occur at the menopausal transition: while T helper 1 (T_H_1) cell/T helper 2 (T_H_2) cell ratios remain relatively stable between pre- and post-menopausal women, aging leads to a pronounced post-menopausal T_H_1 cell bias [18]. The aging pattern of regulatory T (T_reg_) cells is also nonlinear, with a gradual increase in pre-menopause followed by a sharp decrease during the menopausal transition, with a renewed gradual increase in post-menopause [18]. Menopause is therefore a key factor underlying the lack of collinearity in aging trajectories; however, it is hardly ever accounted for.

Menopause onset varies widely. Although the median age at menopause in high-income countries is ~51 years, women with premature ovarian insufficiency (POI) can experience it as early in their twenties, while late menopause can occur up to the early sixties [25–27]. Because of this wide range, aging analyses that do not consider age-at-menopause and the associated hormonal transition introduce uncontrolled, unacceptable heterogeneity, which can either erase true biological signal or lead to overgeneralizations that do not reflect true personal trajectories, thus undermining the goals of precision medicine.

Reproductive aging in men is much more gradual, with gradually declining testosterone levels and reduced fertility [28,29]. Indeed, unlike menopause, which causes abrupt changes, andropause will drive slower, more linear shifts [28]. Starting at age 30, testosterone decreases by ~1% per year, although the rate and physiological impact vary between individuals [28]. Prevalence of hypogonadism prevalence rises from <5% at age 40 to >30% by age 70, reflecting a distinctly nonlinearity of age-related changes [28]. Due to the gradual nature of physiological age-related changes, identifying clinically meaningful testosterone decline is challenging, but its gradually unfolding nature is likely to remain biologically significant, including in its effects on immune aging.

Nonlinear transitions highlight why uniform clinical models fail to capture critical sex-by-age interactions that shape disease susceptibility, therapeutic responses, and overall health outcomes [16,30]. Epidemiological studies, animal research, and clinical trials that merely regress out the effects of age and sex, assuming their additivity and linearity, risk producing misleading conclusions and thus poorly adapted therapeutics [31]. Integrating sex-by-age interactions into study design and analysis is therefore essential for building predictive and pertinent medical protocols.

### Implications for research and medicine

Despite their biological significance, many researchers continue to neglect sex-based analyses, thereby obscuring genuine biological divergence [32]. Historically, many preclinical and clinical studies excluded females or failed to disaggregate data by sex, creating a male-biased evidence base that can result in failed therapeutics or even sex-specific harm [33,34]. Even in diseases that disproportionately affect females, trial designs and dosage standards are still often derived from male-driven data [35,36]. As a result, sex-appropriate dosing, which is critical for minimizing unnecessary adverse effects and improving therapeutic efficacy, is still not the gold-standard [36].

At the preclinical stage, although animal models are informative, they often fail to fully replicate human biology [37]. An important factor may reside in the fact that rodents have very different reproductive aging, experiencing estrus rather than menstrual cycles, and have distinct timing of onset and post-reproductive hormonal landscapes [38,39]. Because complete ovarian failure is a defining feature of human menopause, it is important to note that rodents, while they do experience some ovarian follicle depletion, do not undergo full ovarian exhaustion as humans do [38]. Hormonal cycling itself may affect immune outcomes and could be important to control for [40]. Thus, it is not surprising that mice do not fully reproduce immune and inflammatory profiles seen with human aging [41]. Immune aging research must critically evaluate and refine animal models to make preclinical findings more relevant to human biology, especially as it relates to sex differences [31].

Importantly, even a decade after the implementation of the US National Institutes of Health (NIH) policy on sex as a biological variable, many studies still fail to meaningfully integrate sex-based analyses [42]. Researchers often cite assumptions of phenotypic equivalence between sexes (and thus cost considerations), hormone-driven variability, or X-linked genetic constraints to avoid including females [42]. Together, this pragmatism and the fallacies it is based on have perpetuated a biased framework and distorted models. Indeed, when sex is not properly accounted for, resulting findings cannot be generalized to explain aging or inform therapeutic strategies. Still today, <10% of studies rationalize why females are excluded or why data was not analyzed by sex [32]. Although cases of sex-specific pathology exist and represent a unique exception, interpretability and translatability of research demands new standards where sex must be accounted for in study design; because what is worth doing should be worth doing well.

Even among sex-aware studies, overreliance on linear models that treat age as a continuous variable and sex as a simple categorical factor remain widespread [43,44]. These approaches assume sex differences are constant across life and overlook the key nonlinear, time-dependent interactions that drive biological variation [44]. Significant age–sex interaction terms are often ignored or explained away, as focusing on a single factor can reduce complex biology to statistical convenience [44]. Furthermore, oversimplified analytical models can distort biological interpretation and lead to misleading conclusions [43]. False positives may arise when effects appear significant but are not causal, or when differences attributed to age rather reflect hormonal inflection points [44]. Conversely, false negatives occur when true sex-by-age interactions go undetected in underpowered simplistic models, promoting overgeneralization and obscuring critical periods of vulnerability/resilience [43,44]. Consequently, the complexity of sex-specific aging trajectories and their relevance for personalized treatment remain underappreciated [19]. What is needed is a paradigm shift, whereby such nonlinear interactions—which reflect true biological divergence—become the focus of attention, rather than an afterthought, enabling us to uncover true biological underpinnings of immune aging and to inform tailored, personalized interventions.

To overcome analytical limitations, researchers need models that capture nonlinear immune aging trajectories [45]. Such models, while requiring more statistical proficiency, do exist: mixed-effects models [46,47], generalized additive models (GAMs) [48], and piecewise regression [49] can be used to provide a more realistic view of sex-specific immune aging patterns, which could include modeling of hormonal transitions or sex-specific life history (e.g., past pregnancies and lactation). Mixed-effects models provide a powerful framework to study how sex and aging interact on immune trajectories by simultaneously estimating fixed and random sources of variability [46,47]. Unlike conventional linear models that assume uniform effects, mixed-effects approaches separate variation into consistent and individual-specific components [46,47]. GAMs allow flexible, data-driven relationships, using smooth functions to capture gradual or abrupt biological transitions across the life span rather than forcing linear assumptions [48]. This adaptability makes GAMs especially useful for studying immune aging, where physiological changes are rarely linear, and helps challenge the outdated view that biological change proceeds along a single trajectory and instead portray immune aging as a continuum shaped by nonlinear, sex-specific influences [16]. Finally, piecewise regression can capture sharp biological transitions (e.g., menopause) by dividing data into segments and fitting independent models to detect breakpoints (ages where variable relationships shift significantly) [49]. Together, these nonlinear frameworks have the potential to offer the methodological flexibility required to accurately model and translate biological complexity into actionable therapeutic insights.

## Impact of sex differences immune system aging for health and medicine

There is a consistent theme across infectious diseases, autoimmunity, cancer, vaccine responses, and drug responses: aging and biological sex elicit powerful, intersecting effects on immune function that fundamentally shape health outcomes. These factors do not act independently, but interact to alter adverse event susceptibility, disease risk, and therapeutic benefit, in ways that are predictable yet currently underrecognized in clinical practice. The evidence summarized in the following sections demonstrates that treating age or sex as isolated variables is insufficient for optimizing patient care. Instead, precision medicine approaches that directly integrate both elements are urgently needed to improve diagnosis, prevention, and treatment strategies, ultimately allowing equitable and effective healthcare for everyone.

### Disease incidence and/or severity

The interplay of age and sex differentially impacts infectious disease, autoimmunity, and cancer susceptibility and severity, likely through their effects on the immune system. These interactions are therefore important to consider when assessing disease risk and formulating prevention and treatment plans.

#### Infectious diseases.

The infectious diseases varies considerably as a function of age, with increasing age-associated with increased incidence (in some cases) and severity (in most cases), generally leading to greater susceptibility and higher mortality [50,51]. Immunosenescence contributes to heightened vulnerability and poorer outcomes following infection [52]. In a Danish population study, the incidence of *Staphylococcus aureus* bacteremia increased with age, reaching levels over 10-fold higher in individuals aged 90 or older compared to middle-aged adults (ages 30−50) [51]. Beyond bacterial infections, such age-related increases have also been documented for fungi [53], viruses [54], and parasites [55]. Beyond heightened susceptibility, advancing age is also associated with poorer outcomes of infectious diseases such as COVID-19, malaria, including increased severity, complications, and mortality [50,56,57]. Thus, not only are older adults more likely, in some cases, to contract pathogens that cause infectious diseases, but they are also more likely to experience severe (or fatal) outcomes.

Biological sex also modulates infection incidence and severity [58]. As discussed above, females have a generally more robust immune response compared to age-matched males, leading to male-biased susceptibility and lethality of infections at any age [55,58,59]. The COVID-19 pandemic provided a striking example of this, with males twice as likely to develop severe COVID-19 compared with females [60]. Males are also more prone to sepsis and have poorer outcomes [61,62]. Overall, males are more susceptible to pathogens that cause infectious diseases and have a higher chance of severe consequences, highlighting the importance of integrating sex as an important variable in public health and clinical interventions.

While age and sex both impact infection susceptibility and severity, neither of these factors acts in isolation [63]. Indeed, interactions between age and sex profoundly influence both susceptibility to and severity of infectious diseases [59,61]. For example, listeriosis and tick-borne encephalitis are more frequent in men compared with women, but only after the age of 60 [59]. Additionally, women with sepsis had shorter hospital stays and lower mortality compared to men, but only after the age of 44 and 47, respectively [61]. Such breakpoints in the emergence of sex differences can be the result of ovarian aging: menopause occurs earlier and results in a sharp hormonal decline than andropause [64], and menopause leads to increased infection susceptibility [65]. Thus, the immune advantage females have at younger ages may shrink or be lost altogether with advancing age [66].

#### Autoimmune diseases.

Aging has a crucial role in shaping autoimmunity risk, whereby advancing age is associated with increased incidence of autoimmune diseases [67–70]. Inflammaging itself can create an environment conducive to developing autoimmune diseases [7]. For example, the prevalence of rheumatoid arthritis increases with age across populations [71,72]. Sex is also a major driver of autoimmunity, with females accounting for ~60%–70% of patients diagnosed with autoimmune diseases [69,70]. In fact, females account for ~90% of individuals with specific autoimmune diseases (e.g., Sjögren’s syndrome, systemic lupus erythematosus; SLE) [69]. However, despite the female bias in autoimmune risk, some autoimmune conditions are more common in males (e.g., inclusion body myositis, acquired hemophilia, and ankylosing spondylitis) [69,73]. Inherently stronger immune responses in females are likely to blame for the overall female bias in autoimmunity [58].

Taken together, sex and aging are major factors in shaping autoimmunity risk, where they have both interacting and independent influences [70,74]. For example, a German cross-sectional analysis of 31 different autoimmune diseases found that, although autoimmune disease prevalence increased with age in both sexes, it peaked at different ages (75–79 years in women, 80–84 years in men) [70]. Similarly, elderly-onset SLE, which predominantly affects females, appears to be driven in part by menopause, accentuating the role of sex hormones in shaping immune system function and influencing age- and sex-specific disease risk [74]. Thus, it will be crucial to devise tailored prevention and treatment strategies for older individuals with autoimmune diseases.

#### Cancer.

Aging is the biggest risk factor for developing cancer [75]. Indeed, cancer incidence rises sharply with age, from <26 cases per 100,000 in individuals aged under 20, to ~350 per 100,000 among those aged 45–49, and >1,000 per 100,000 in those aged over 60 [76]. Reflecting the role of age as a risk factor for most cancer types, the median ages at diagnosis are 71 years for lung cancer, 73 years for bladder cancer, and 68 years for stomach cancer [77,78]. Mechanistically, multiple aging hallmarks can regulate cancer initiation and tumor progression, including progressive telomere shortening and DNA damage accumulation [77,79]. These cell-intrinsic age-driven changes are also compounded by an age-related decline in immune surveillance and effectiveness, reducing the host’s ability to detect and eliminate emerging tumor cells. Sex is also a major influence in incidence of specific cancers [58,80]. While reproductive cancers are a trivial example of sex-biased cancers (e.g., prostate or ovarian cancer) [81,82], biological sex (as well as gender-driven exposures) also influence cancer incidence and mortality of nonreproductive cancers [58,80]. Indeed, males exhibit higher incidence and age-adjusted mortality rates for most cancers (e.g., male-to-female mortality rate ratios are 5.51 for lip cancer, 4.08 for esophageal cancer, and 3.36 for urinary bladder cancer) [80]. Male-biased mortality is thought to arise from sex differences in cancer etiology, including variations in viral susceptibility, immune responses, gene expression, hormonal regulation, sex chromosome karyotype, autophagy, oxidative stress, or a combination of these factors [58,80].

In addition to their independent impacts, age and sex also have nonlinear impacts on cancer incidence and mortality [83–85]. For example, in a pan-cancer analysis study, men exhibited higher cancer incidence across nearly all sites and age groups, aside from individuals aged 20–39 years, among whom women showed higher incidence across all primary cancer sites [83]. A similar pattern was observed in primary lung cancers, with women’s incidence rates initially being higher at ages 20–49, before a gradual risk reversal towards male-biased incidence with increasing age, and became male-biased by age 80 and older [85]. Although not directly examined in these studies, the timing of this shift in incidence roughly overlaps typical age-at-menopause, suggesting that ovarian hormone influences on immune function could contribute to the age- and sex-differences [19].

### Vaccine immunogenicity and adverse events

Vaccine immunogenicity (Box 1) markedly decreases with aging [86,87]. A meta-analysis of SARS-CoV-2 vaccine responses revealed that younger adults had significantly higher geometric mean antibody titers (GMTs) than older adults, indicating that they had greater vaccine-induced immunogenicity [86]. Multiple studies have also shown that sex shapes vaccine-induced immune responses [58,88]. For instance, a meta-analysis of 18 studies on the quadrivalent human papillomavirus (HPV) vaccine revealed that females exhibited higher GMTs across all age groups [88]. Sex differences in vaccine immunogenicity are thought to arise from a combination of factors, including heightened antibody production and T cell cytotoxicity in females, genetic influences, and hormonal regulation of immune cell function [88]. Overall, vaccine-induced immune responses are sculpted by both age and sex. Younger individuals and females generally mount stronger antibody responses across multiple vaccines, highlighting the importance of considering these factors in vaccine research, tailoring immunization schedules, and informing public health recommendations.

Akin to vaccine immunogenicity, adverse events following vaccination are also influenced by age, with younger individuals typically experiencing a higher incidence than older adults [86,89,90]. In terms of vaccine safety, the meta-analysis described above reported that younger individuals experienced a higher incidence of both local and systemic adverse events following SARS-CoV-2 vaccination, likely reflecting a stronger immunity in younger individuals and immune decline in older adults [86]. Similarly, sex potentially also affects adverse events, with women reporting a higher incidence of adverse events than men [91–93]. This phenomenon is highlighted in a study of the Pfizer-BioNTech SARS-CoV-2 vaccine, where women had a higher risk of local, systemic, and sensory adverse events across age groups, likely caused by their overall robust immune responses [58,93]. Importantly, observed sex differences in adverse event reporting may partly reflect behavioral differences, with males less likely than females to report mild to moderate events, though this remains to be formally tested [92]. Vaccine-related adverse event risk is shaped by age and sex, with younger individuals and females typically at higher risk, stressing the need to consider these factors in vaccine safety assessments and immunization strategies.

The interaction of age- and sex-related immune differences also impacts vaccine immunogenicity and adverse events; SARS-CoV-2 vaccination provides a clear example of how age and sex interact to modulate immune responses [94]. A longitudinal study investigating how age and sex influence immune responses to SARS-CoV-2 vaccination in older adults found a sex-specific effect of aging, with males displaying a greater age-associated decline in humoral immunity than females [94]. Consequently, the difference in antibody titers between males and females widened with age [94]. Importantly, booster vaccination restored antibody responses and eliminated sex- and age-related disparities, showcasing why both factors should be considered when guiding clinicians and shaping public health vaccination recommendations for older adults [94]. The interaction between age and sex also has a critical role in molding the incidence and severity of vaccine-related adverse events [95]. In Thailand, a study of serious adverse effects after SARS-CoV-2 booster vaccination reported that age and sex cooperatively influenced the likelihood of these events, as identified by clinicians [95]. Specifically, the risk of serious adverse events was higher in females aged 12–40 versus age-matched males, while males aged over 50 exhibited a higher risk than age-matched females [95].

Influenza similarly demonstrates how age and sex interact to shape immune responses [96]. A meta-analysis of phase 3 trials (2010–2018) found that females showed increased influenza vaccine immunogenicity, although this was only observed in individuals over 65 years of age [96]. There is also evidence of interaction between sex and age in adverse events associated with influenza vaccination [97]. Indeed, a 2020 study found that peak incidence occurs at substantially different ages in females (~53 years) compared with males (during infancy) [97].

Pneumococcal pneumonia is another example illustrating how age and sex impact vaccine immunogenicity [98]. Indeed, in a study evaluating pneumococcal vaccine effectiveness in older adults using systems immunology, females mounted stronger immune responses to the conjugated PCV13 vaccine, while there were no sex differences for the unconjugated PPSV23 vaccine [98]. The authors identified an immune phenotype, prevalent in older males, which was associated with weaker PCV13 responses, characterized by increased numbers of CD16⁺ natural killer cells and IL-17-producing helper T cells, fewer T_H_1 cells and increased cytotoxic gene expression, which may underlie reduced vaccine responsiveness in older males compared with females [98]. The impact of age–sex interactions on pneumococcal vaccine adverse event risk remains poorly understood.

Together, these studies emphasize the importance of integrating information on sex differences in immune system aging to optimize vaccine responses and tailor safety recommendations to inform public health strategies and clinical practice. One important note is that while we have described many cases of vaccine adverse events being modulated by age and/or sex, vaccines rarely cause life-threatening conditions and remain the safest and most effective intervention to prevent severe outcomes from infectious diseases [99].

### Cancer treatment efficacy and adverse events

Immunotherapy is a form of cancer treatment that modulates the body’s own immune system to be able to recognize and neutralize cancer cells [100]. Interestingly, the efficacy of immunotherapy is heavily influenced by aging [101,102]. Studies indicate that patients up to ~70 years of age experience consistent survival benefits from immunotherapy [102], whereas those >75 years of age derive less benefit and have lower survival improvements [101]. While there is reduced efficacy in individuals aged >75, immunotherapy still provides a meaningful reduction in mortality risk in this age group, indicating that immunotherapy is beneficial but may require adjustments based on age [101]. Unfortunately, according to data from retrospective studies, aging also leads to heightened risk for immune-related adverse events (irAEs) [103,104]. Interestingly, upregulation of JNK cascade-related genes and collagen extracellular matrix components in myeloid cells may contribute to this heightened irAE risk in older adults [104]. Overall, these studies show that, since advanced age leads to decreased efficacy and increased risk of adverse events, age-specific approaches are likely required to optimize care [101,103].

Consistent with the existence of sex differences in the aging immune system, sex also substantially impacts immunotherapy efficacy and risk [105,106]. A systematic review and meta-analysis of 20 randomized controlled trials of immune checkpoint inhibitors found that survival was greater in male versus female patients (28% versus 14% reduction in the risk of death) [105]. Consistently, females receiving immunotherapy had a 49% higher risk of severe adverse events [106]. Potential contributors to sex differences in severe adverse events include sex differences in drug metabolism/clearance rates, which are well documented and are believed to drive most sex-biased drug adverse effects [107]. These findings underscore the need for greater female representation—and sex-segregated reporting—in immunotherapy clinical trials and for careful consideration of biological sex when recommending and administering these treatments, with strategies tailored to optimize efficacy and minimize adverse events for each sex [105,106].

While many studies stratify immunotherapy efficacy and adverse event rates by age or sex independently, there is a dearth of research examining how these two factors interact. One study examined how both age and sex influenced immune checkpoint kinetics and expression in human T cells and suggested that older females showed a more pronounced decline in PD-1⁺ memory CD4⁺ T cells compared with older males, which could decrease efficacy from immune checkpoint inhibitor therapy [108]. Thus, further research will be needed in this area at both the clinical and preclinical levels.

### Clinical pharmacology, drug response, and adverse events

Clinical pharmacology (Box 1) encompasses pharmacokinetics (how the body processes drugs) and pharmacodynamics (how drugs affect the body) [109], both of which are impacted by aging [110]. Pharmacokinetically, renal and hepatic clearance decline with age and distribution volume for lipid-soluble drugs increases, prolonging drug half-life (leading to potential overdose risk) [110]. Pharmacodynamically, older adults often show heightened sensitivity to many drug classes, including anticoagulants, psychotropics, and cardiovascular agents [110]. One potential contributor to this phenomenon is polypharmacy, the concurrent use of several medications, which is more common in older individuals [111]. Combined with age-related pharmacokinetic and pharmacodynamic changes, polypharmacy can reduce drug efficacy and increase the risk of adverse events, underscoring the need for age-appropriate drug selection and dosing to maintain a favorable benefit-risk balance [110–112].

**Fig 2:**
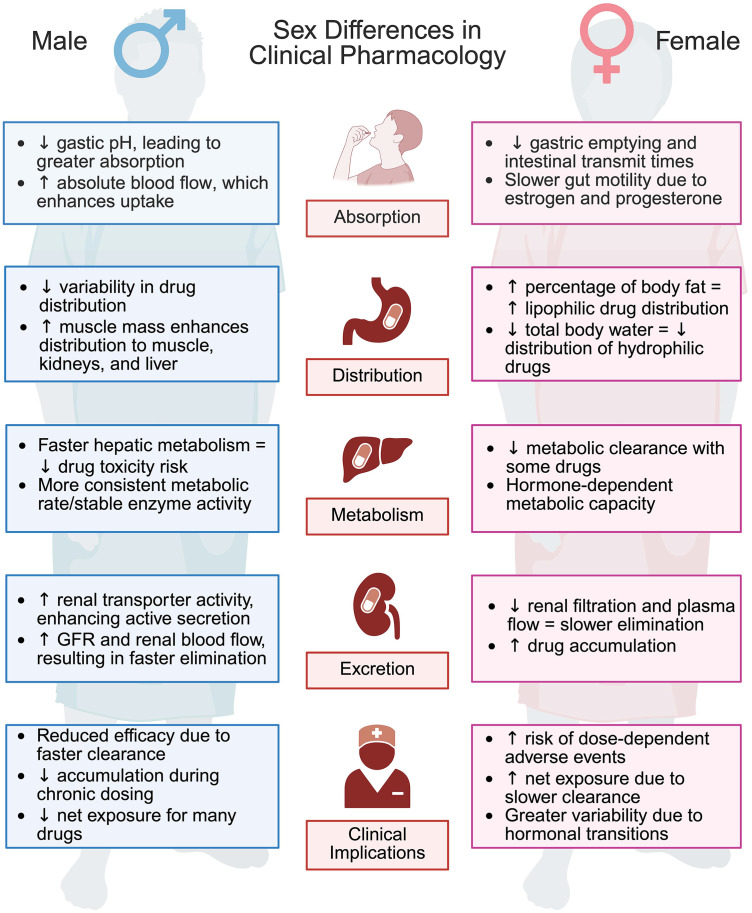
Sex differences in clinical pharmacology. Biological sex influences all phases of clinical pharmacology. During drug absorption, males generally exhibit faster uptake due to lower gastric pH and higher absolute blood flow, whereas females tend to have slower absorption associated with delayed gastric emptying and reduced gut motility. Sex differences also shape drug distribution: males show less inter-drug variability and greater distribution to muscle, kidney, and liver, largely reflecting higher lean muscle mass. By contrast, females exhibit enhanced distribution of lipophilic drugs due to a higher proportion of body fat, but reduced distribution of hydrophilic drugs because of lower total body water. Drug metabolism further diverges by sex, with males displaying faster hepatic metabolism and more consistent enzyme activity, while females have reduced clearance of certain drugs, often in a hormone-dependent manner. Differences in drug excretion compound these effects: males typically have enhanced renal elimination driven by higher glomerular filtration rate (GFR), renal blood flow, and transporter activity, whereas females experience slower elimination and greater drug accumulation due to lower renal filtration and plasma flow. Collectively, these sex-specific differences have important clinical implications. Faster clearance in males often results in reduced drug exposure and efficacy, particularly during chronic dosing, whereas slower clearance in females increases net exposure and the risk of dose-dependent adverse events. This figure was created using BioRender. Baker, C. (2025) https://BioRender.com/uw5c86p.

Pharmacokinetics is also greatly affected by biological sex [36]. In a literature review of 86 drugs, it was observed that for most FDA-approved drugs examined, females exhibited higher blood concentrations and longer elimination times, and these pharmacokinetic differences were closely associated with their greater susceptibility to adverse drug reactions (ADRs) [36] (Table 1). Importantly, sex differences in pharmacokinetics were not explained by sex differences in body weight [36]. Interestingly, while 96% of drugs with pharmacokinetics biased toward females were linked to a higher incidence of ADRs, only 29% of drugs with male-biased pharmacokinetics predicted a higher incidence of ADRs [36]. Overall, these results provide clear evidence that biological sex must be considered in drug-dosing, as a one-size-fits-all approach can be detrimental to half the population, underscoring the need for more personalized dosing strategies [36] (Fig 2).

**Table 1:** Drugs withdrawn or relabeled due to female-biased toxicities.

Drug	Prescribed for	Female-biased adverse events	Year of approval	Regulatory action	Reference(s)
Alosetron	Irritable bowel syndrome with diarrhea	Serious gastrointestinal events occurred mainly in women, including severe constipation and intestinal ischemia	2000	Withdrawn in 2000, then reintroduced in 2002 with restrictions	[113,114]
Astemizole	Seasonal allergies/hay fever	Women experienced more life-threatening heart rhythm abnormalities	1981	Withdrawn in 1999	[113]
Cisapride	Acid reflux/delayed stomach emptying	Women accounted for most severe arrhythmia cases	1993	Withdrawn in 2000	[113]
Dexfenfluramine	Weight management/obesity	Women had higher rates of heart valve disease	1996	Withdrawn in 1997	[113]
Fenfluramine	Weight management/obesity	Most early heart valve problems reported in women	1973	Withdrawn in 1997	[113]
Mibefradil	High blood pressure/angina	Lowered heart rate in elderly women	1997	Withdrawn in 1998	[113]
Terfenadine	Seasonal allergies/hay fever	Women had higher incidence of ventricular arrhythmias	1985	Withdrawn in 1997	[113]
Troglitazone	Type 2 diabetes	Women accounted for more than two-thirds of the deaths due to liver failure	1997	Withdrawn in 2000	[113]
Zolpidem	Insomnia/sleep aid	Higher risk of next-morning impairment in women at standard dose	1992	Label updated in 2013 to recommend lower dose for women	[115–117]

While age and sex independently modulate clinical pharmacology, there is also evidence that their interaction can profoundly shape pharmacokinetics [118]. In a systematic review, females were shown to have an age-related increase in median peak drug concentrations compared with males [118]. Notably, this relative age-related increase in plasma concentrations in females could underlie the greater age-associated rise in adverse event risk observed versus males [118]. Therefore, there is convincing evidence that both age and sex should be incorporated into drug-dosing regimens to reduce the burden of ADRs, particularly in older females.

Immunomodulatory drugs are also influenced by sex and age. Nonsteroidal anti-inflammatory drugs (NSAIDs) are a class of drugs used to decrease inflammation, relieve pain, and reduce fever (e.g., ibuprofen, naproxen) [119]. Interestingly, NSAID-associated adverse events are influenced by aging [120]. In a retrospective analysis of NSAID use in Japan, individuals aged ≥65 had higher incidences of gastrointestinal (GI), renal, and acute myocardial infarction events associated with NSAIDs (compared to younger individuals), which were further exacerbated in individuals aged >80 [120]. Similarly, biological sex also impacts NSAID-associated adverse events [121]. Results from a nested case–control study showed that females were about twice as likely as males to experience serious NSAID-related GI adverse events [121]. This disparity is particularly striking given that males typically have a 1.4-fold higher baseline risk of serious GI events [121]. Thus, sex should be a key factor in NSAID prescribing and dosing decisions. Importantly, evidence also shows that age and sex interact on NSAID-related adverse events risk [122]. A prospective cohort study of >130,000 individuals found that acute kidney injury (AKI) was higher in males than females, and that the relative risk for males further increased with age [122]. Of note, the study also found that females aged >55 who received estrogen therapy had a lower risk of AKI compared to age-matched females not receiving estrogen therapy, suggesting that gonadal hormones and menopause may modulate this risk [122].

Prednisone and prednisolone are glucocorticoid drugs (also known as corticosteroids) that are used as potent immunosuppressant and anti-inflammatory agents [123], the efficacy and adverse event risk of which are greatly influenced by aging [124,125]. One study found that although elderly individuals had higher plasma prednisolone levels, they exhibited weaker plasma cortisol suppression, suggesting reduced efficacy compared with younger individuals [124]. Another study found that among individuals taking prednisolone, aging was associated with a significant increase in hip fractures in both sexes, with this effect being markedly stronger in females than in males [125]. Biological sex also clearly influences the risk of adverse events associated with prednisone/prednisolone [126]. In a survey study on prednisone use for myasthenia gravis, an autoimmune disorder, adverse events were reported more frequently in females than males (95% versus 81%) [126]. Females also reported more intolerable adverse events (77% versus 50%) and were less willing to accept a dose increase (26% versus 44%), suggesting a worse safety profile [126]. Evidence shows that sex and age also interact to influence prednisone efficacy [127]. A retrospective observational study found that older age at the start of prednisone treatment for inflammatory bowel disease was associated with reduced treatment efficacy, but only in females [127].

Tumor necrosis factor (TNF) modulators are biological drugs that specifically target the TNF protein, a key mediator of the inflammatory response [128]. A comprehensive literature review found that older patients experienced poorer efficacy outcomes from anti-TNF agents in 5 out of 6 studies examined [129]. In addition to reduced efficacy of TNF modulators in older individuals, a separate study of patients with rheumatoid arthritis receiving anti-TNF therapy found that older patients experienced more adverse events [130]. The efficacy of TNF modulators is also influenced by sex [131]. In a pooled observational study of patients with psoriatic arthritis receiving TNF inhibitors, females were ~18% less likely than males to achieve low disease activity [131]. Interestingly, females also had significantly lower treatment retention, potentially reflecting a higher risk of adverse events [131]. Supporting a nonlinear interaction of age and sex, a retrospective analysis of patients with rheumatoid arthritis treated with TNF inhibitors found that secondary nonresponse occurred in both males and females, but the age at which nonresponse developed differed by sex (females at 60–81 years, males at 40–59 years) [132]. Together, these examples show that benefit-risk equation for many immunomodulatory drugs is likely different as a function of patient sex and age.

## Conclusions and future directions

The historical exclusion of females from clinical research continues to limit our understanding of immune aging and its biological variability [133]. In response to thalidomide-related pregnancy tragedies, the US Food and Drug Administration issued its 1977 guideline “General Considerations for the Clinical Evaluation of Drugs”, which unintentionally created a male-biased research standard (Box 2), obscuring how processes such as immune aging differ by sex or age, or by the interaction of these two factors [134–137]. As a result, key aspects of female immune aging, such as menopausal transition, remain poorly characterized, hindering progress towards personalized medicine.

**The 1977 FDA recommendation and its impact on the health of females.**

In the late 1950s and early 1960s, the use of thalidomide for pregnant females caused thousands of infants worldwide to be born with severe limb deformities and led to countless deaths [137]. The United States avoided the worst of this tragedy due to the US Food and Drug Administration’s (FDA) insistence on rigorous scientific evidence before drug approval [138]. The Kefauver–Harris Amendments, passed by Congress in 1962, gave the FDA the power to demand evidence of a drug’s effectiveness and to mandate that unexpected adverse events be reported [137].

Despite these reforms, additional events throughout the 1960s and 1970s, such as complications associated with high dose estrogen contraceptives, and the discovery that prenatal exposure to diethylstilbestrol increased the risk of clear cell vaginal adenocarcinoma, renewed concerns about drug safety and reproductive toxicity [137]. In response to these tragedies, the FDA issued the 1977 guidance “General Considerations for the Clinical Evaluation of Drugs”, which recommended excluding females of childbearing potential from Phase 1 and early Phase 2 clinical trials, except in life-threatening conditions [135,137]. The term ‘childbearing potential’ was generally interpreted to include nearly all premenopausal females, regardless of abstinence or contraceptive use [135,137,139].

Although intended to protect females and potential fetuses from harm, the policy had unintended consequences [135]. By systematically excluding females from early-stage clinical research, it delayed understanding of sex-specific drug metabolism, efficacy, and toxicity [135]. This oversight contributed to decades of male-biased dosing standards and a higher incidence of adverse drug reactions in females [135]. The 1977 policy thus represents a pivotal moment in regulatory history, one that underscores the necessity of balanced inclusion, transparency, and sex-aware study design to achieve equitable and effective medical care [135].

Federal policies since then have increasingly emphasized the ethical and scientific necessity of including women in clinical research [42]. These efforts culminated in the US NIH Revitalization Act of 1993, which mandated the inclusion of women in federally funded clinical research, and were further strengthened by the 2016 NIH Sex as a Biological Variable policy [139].

Progress began with the NIH Revitalization Act of 1993, which required the inclusion of females and individuals from minority groups in federally funded research [140]. Yet females and individuals from other minorities remained underrepresented in aging studies, including vaccine and immune trials [140]. The 2016 NIH Sex as a Biological Variable policy emphasized that sex should be treated as a fundamental biological factor at all stages of research, from in vitro systems to clinical populations [42].

**Fig 3:**
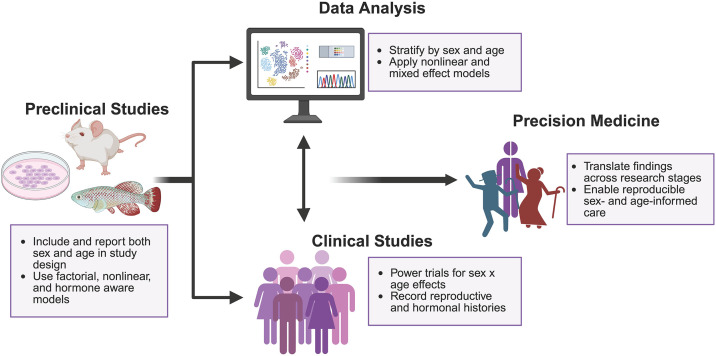
Considerations for sex- and age-informed personalized medicine. Overview of how sex and age can be incorporated across different research stages, from preclinical models through data analysis and clinical studies, to inform precision and personalized medicine. In preclinical studies, inclusion of both sex and age, along with factorial and nonlinear designs improve biological interpretability. Analytical approaches that stratify by sex and age and apply nonlinear and mixed-effects models allow detection of age-dependent and sex-specific patterns. In clinical studies, trial designs powered to detect sex-by-age interactions and systematic recording of reproductive and hormonal histories support more informative outcome and interpretation. Together, these considerations make it easier to translate study findings and make sex- and age-informed care more reproducible and useful. In the “preclinical studies” (left), a mouse, killifish, and a plate of cells are depicted. Following this, the “data analysis” (top middle) is represented through machine learning and “clinical studies” (bottom middle) shows a group of individuals representing both sexes, with different colors used to indicate diverse backgrounds. For “precision medicine,” (right) there is an old male and female, with the young male and female split in half behind them. This figure was created using BioRender. Baker, C. (2025) https://BioRender.com/64fqlw8.

Similarly, in Europe, the consideration of sex as a biological variable has become important to research policy. Within the Fifth Framework Programme for Research and Technological Development (FP5) and subsequent EU Framework Programmes, studies emphasized the need to clearly define sex and incorporate sex-based analysis into health and life sciences research [141]. Building on these efforts, the European Commission promoted both increased participation of women in research and the integration of sex-based considerations into research design [141]. Consequently, applicants to EU-funded programmes are expected to address sex-related aspects in their proposals, and these considerations form part of the evaluation criteria for funding [141]. However, most studies still fail to integrate age as a dynamic co-variable, neglecting how immune function evolves across the life span in sex-specific ways [16,142]. For successful personalized medicine, sex, and age must be treated as interdependent biological variables (Fig 3), not as optional considerations or annoying confounding variables, even in vitro. Researchers should record the sex chromosome karyotype of cell lines, include male and female cells, and document relevant culture conditions that affect outcomes [143]. For instance, including hormones such as estrogen or testosterone, when appropriate, can reveal hormone-dependent mechanisms that shape immune function and aging trajectories [31].

Preclinical animal studies should also systematically include both sexes and explicitly consider age-related variability [143]. The sex and age of all animals should be clearly reported, with reports also segregating (or at least clearly annotating) data from each sex separately. Factorial or stratified designs are essential to distinguish independent and interactive effects of sex and age [144]. Nonlinear approaches, such as mixed-effects models, GAMs, and piecewise regression, can capture inflection points and transitions that linear models miss, providing a more accurate view of immune aging [46,48,49]. To appropriately model hormonal transitions and the impacts thereof on immune aging and disease, accumulating models are being characterized and are showing their potential [31,145]. Including such models could increase the translatability of preclinical findings outside of the laboratory.

Clinical trials must also be intentionally powered and designed to detect sex-by-age interactions [143]. Recruitment strategies should ensure balanced representation of females and older adults, while comprehensive reproductive and hormonal histories including age at menarche and menopause, pregnancy outcomes, hormonal contraceptive use, and hormone therapy should be well documented [143]. For premenopausal females, menstrual cycle phase, sex-specific biomarker thresholds, and pharmacogenomic differences must be considered [18]. Stratifying data by both sex and age will reveal immunological and pharmacologic differences that combined analyses routinely conceal [118]. Even after drug approval, continuous oversight is essential [146]. Post-marketing surveillance should be transparent and rigorously assess interaction effects, with findings reverse-translated into basic research to reveal the underlying biological mechanisms [146]. Only by embracing the nonlinear dynamics of immune aging can science achieve sex- and age-informed precision medicine with reproducible, interpretable, and translatable results.

## Abbreviations:

ADRs: adverse drug reactions
AKI: acute kidney injury
DCs: dendritic cells
FDA: Food and Drug Administration
GAMs: generalized additive models
GFR: glomerular filtration rate
GI: gastrointestinal
GMTs: geometric mean antibody titers
HPV: human papillomavirus
irAEs: immune-related adverse events
NIH: National Institutes of Health
NSAIDs: nonsteroidal anti-inflammatory drugs
POI: premature ovarian insufficiency
T_H_1: T helper 1
T_H_2: T helper 2
TNF: Tumor necrosis factor
T_reg_: regulatory T.
